# Improved Quantification of ICG Perfusion Through Motion Compensation in Fluorescence-Guided Surgery

**DOI:** 10.3390/diagnostics16020176

**Published:** 2026-01-06

**Authors:** Sermed Ellebæk Nicolae, Thomas Baastrup Piper, Nikolaj Albeck Nerup, Michael Patrick Achiam, Morten Bo Søndergaard Svendsen

**Affiliations:** 1 Department of Digestive Diseases, Transplantation and General Surgery, Copenhagen University Hospital Rigshospitalet, Blegdamsvej 9, 2100 Copenhagen, Denmark; sermed.ellebaek.nicolae@regionh.dk (S.E.N.); thomas.baastrup.piper.02@regionh.dk (T.B.P.); nikolaj.albeck.nerup@regionh.dk (N.A.N.); michael.patrick.achiam.01@regionh.dk (M.P.A.); 2 Department of Clinical Medicine, University of Copenhagen, 2200 Copenhagen, Denmark; 3 Drug Delivery and Sensing, DTU HealthTech, Technical University of Denmark, 2800 Kongens Lyngby, Denmark

**Keywords:** motion compensation, motion artifacts, fluorescence-guided surgery, indocyanine green, quantification of indocyanine green

## Abstract

**Background/Objectives**: Motion artifacts significantly distort fluorescence measurements during surgical perfusion assessment, potentially leading to incorrect clinical decisions. This study evaluates the efficacy of automated motion compensation (MC) in quantitative indocyanine green (q-ICG) imaging to improve the accuracy of perfusion assessment. **Methods**: Frames from ICG perfusion assessment during 17 pancreaticoduodenectomies were analyzed. Regions of interest (ROIs) were systematically placed on each frame series, and automated MC was applied to track tissue movement. Performance was evaluated by comparing MC with surgeon-adjusted placement using multiple image quality metrics and analyzing perfusion metrics on time–intensity curves. Principal Component Analysis (PCA) was applied to explore whether image patterns could distinguish between successful and unsuccessful motion compensation. **Results**: Automated motion compensation successfully corrected motion artifacts in 67.5% of frame sequences, achieving comparable performance to surgeon-guided adjustments. PCA demonstrated clear separation between sufficient and insufficient corrections (AUC = 0.80). At the population level, MC did not significantly change perfusion slope (*t*(59) = 1.60, *p* = 0.11) or time-to-peak (Tmax; *t*(58) = 0.81, *p* = 0.42). Bland–Altman analysis showed a mean bias of −0.54 (SD = 3.32) for slope and 24.95 (SD = 238.40) for Tmax. At the individual level, 86.7% of slope and 79.7% of Tmax values differed by ≥10% after MC, with mean absolute percentage changes of 108.5% (median 37.8%) and 431.5% (median 65.9%), respectively. **Conclusions**: MC effectively reduces motion artifacts in fluorescence-guided perfusion assessment. By improving the precision of ICG-derived parameters, this technology enhances measurement reliability and represents an enabler for accurate intraoperative perfusion quantification.

## 1. Introduction

Intraoperative perfusion assessment plays a pivotal role in surgical safety and remains a significant clinical challenge [1]. Inadequate perfusion contributes to serious complications, such as anastomotic leakage and ischemia, with leak rates ranging from 1 to 13% after gastrointestinal surgery and associated 30-day mortality of 8.4% [2]. The adoption of minimally invasive approaches has further challenged traditional assessment methods such as palpation and visual inspection, increasing reliance on subjective judgment that may result in inadequate resections [3].

Indocyanine green (ICG) fluorescence-guided surgery has become a promising technology for real-time perfusion visualization [4,5,6]. It decreases anastomotic leak rates in colorectal surgery [7,8,9] and enhances outcomes in gastric cancer surgery [10] by providing surgeons with improved assessment capabilities beyond what is visible with white light. When given intravenously, ICG binds to plasma proteins and remains intravascular, allowing visualization of perfusion of tissue [11]. However, ICG is susceptible to photobleaching, where prolonged exposure to excitation light leads to a progressive decline in fluorescence signal, which may compromise the consistency of repeated measurements. This can further contribute to variability in fluorescence-based assessments [12]. While qualitative fluorescence assessment provides valuable real-time visualization, it remains inherently subjective and dependent on factors such as camera distance, exposure, and observer experience, resulting in considerable interobserver variability [13,14].

Recent developments in quantitative ICG analysis (q-ICG) enable objective assessment by analyzing fluorescence intensity over time within defined regions of interest (ROIs) [15,16,17,18]. This technique transforms visual impressions into numerical perfusion parameters, which have been shown to correlate to postoperative outcomes, particularly anastomotic healing and tissue viability [19,20,21,22,23]. Quantitative approaches may therefore provide more consistent data [17,18] and facilitate interobserver reproducibility [13,14]. Quantitative ICG assessment represents an evolution from qualitative fluorescence imaging that may facilitate perfusion assessment across different levels of surgical experience and help novices and experienced surgeons in proper, reproducible assessment [15]. While experienced ICG users can visually recognize gross hypoperfusion, subtle differences in perfusion dynamics are easily missed or inconsistently interpreted. Standardized quantitative parameters could therefore complement surgical judgment by providing objective support [15]. The reliability of these parameters, however, depends on maintaining spatial alignment between ROIs and the underlying tissue throughout imaging. A key issue with quantification of ICG is motion interference, which is not visible to the naked eye but appears clear on perfusion curves, and is caused by handheld camera movement. Mechanical disruptions and patient tissue motion (e.g., from breathing, pulsation or peristalsis) result in erroneous measurements that can lead to incorrect time–intensity curves and thus an incorrect presentation of tissue perfusion. For q-ICG to provide reliable data, it requires relatively static imaging conditions that are difficult to maintain in the dynamic surgical environment [17]. Motion compensation (MC) technology addresses this challenge by automatically tracking tissue movement and adjusting measurement regions accordingly. This computational approach maintains ROI positioning on the target tissue despite physiologic and external movements, potentially providing the surgeon with more accurate and reliable perfusion data. So far, quantitative analysis has been proven to have a clinical impact in colorectal surgery [22], but has yet to be evaluated in other branches of surgery. By accommodating for inevitable movement in the surgical field, MC could significantly enhance the clinical applicability of q-ICG across a broad range of procedures.

The present study evaluated the efficacy of automated motion compensation compared to surgeon-adjusted motion compensation in q-ICG perfusion assessment during pancreaticoduodenectomy. We specifically examine how MC affects key perfusion parameters that may be used to guide surgical decision-making and establish thresholds for its successful application in clinical practice. By addressing the critical limitation of motion artifacts, this study aims to enhance the reliability and clinical utility of q-ICG in terms of tissue viability assessment as an intraoperative adjunct.

## 2. Materials and Methods

### 2.1. Imaging Data

This study utilized video recordings from an Institutional Review Board-approved trial conducted in our department during 2022–2023. The trial enrolled 100 patients undergoing pancreaticoduodenectomy with ICG perfusion assessment of the pancreaticojejunostomy to evaluate associations between anastomotic perfusion and postoperative leakage. A total of 17 random patients of the original 100 were included to ensure enough data frames to detect a difference between the MC-reviewed recordings and the ones without MC. Investigators remained blinded to the main study, and no recordings were preselected. Only a subset of recordings was included due to the large effort for manual annotation described later in the methods section. This study of pancreaticoduodenectomies was chosen due to the motion disturbances in the recordings, despite the fixation of the camera, and not due to the specific procedure or branch of surgery. Additionally, this procedure involves imaging of multiple tissue types (pancreas, jejunum, liver) with different perfusion characteristics, allowing for comprehensive assessment of the motion compensation technique in one field of vision.

All ICG assessments and recordings were performed with the optical 4K visualization system SynergyID (Arthrex Inc., Naples, FL, USA). Recordings and images were captured with a 30-degree-angle laparoscope. Standard ICG dosing of 0.2 mg/kg Verdye (Diagnostic Green GmbH, Aschheim-Dornach, Germany) [24] was administered intravenously and flushed with saline, the ventilator was briefly set on hold during recording, and imaging was conducted for up to approximately 150 s after ICG administration to capture both the arterial and venous phases of perfusion.

The study was conducted in accordance with the Declaration of Helsinki and approved by the Institutional Review Board The study was approved by the local Institutional Review Board of Rigshospitalet on the 12 January 2023 (ID-no.: 22003091). The study was conducted on retrospective data, prospectively obtained for clinical quality improvement purposes. Exemption from ethical approval and informed consent was granted from the Institutional Review Board of Rigshospitalet. All patients were presented with the choice to not participate in the quality improvement study. The study complies with all national laws on data protection.

### 2.2. Applications of Quantitative ICG Assessment (q-ICG)

**Figure 1:**
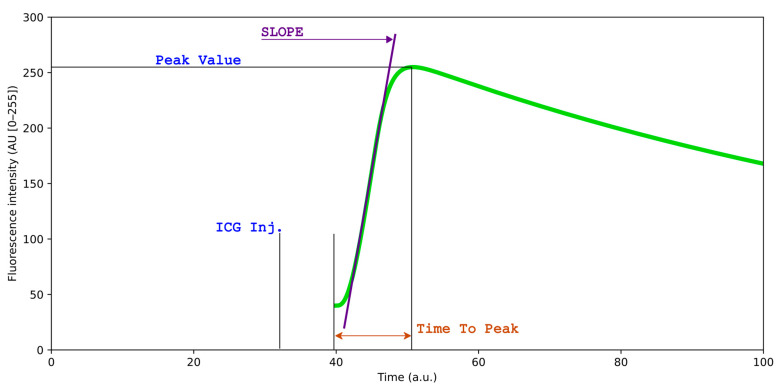
Simulated time–intensity curve of indocyanine green (ICG) fluorescence perfusion, for illustrative purposes. The curve illustrates the dynamic changes in fluorescence intensity over time following ICG administration, reflecting tissue perfusion characteristics. Slope ingress, peak intensity (Tmax), and time-to-peak provide quantitative metrics for perfusion analysis.

Q-ICG methodology, previously used in multiple clinical and pre-clinical studies [15,16,17,18], enables objective measurement of tissue perfusion by tracking the fluorescent signal intensity of ICG over time. Measurement is accomplished by placing ROIs on target tissues and measuring the mean pixel intensity within these regions across sequential video frames. The resulting time–intensity curves reflect the wash-in and wash-out kinetics of ICG, which directly correlate with tissue blood perfusion (Figure 1) [18]. Q-ICG can be performed postoperatively using video recordings, but real-time q-ICG is also feasible and easy to use intraoperatively. When ICG is administered, q-ICG will present the surgeon with a time–intensity curve of the fluorescent signal and various quantitative parameters. In clinical application, these curves can be translated into a relative perfusion score (0–100) in each ROI compared with normoperfused tissue for guidance of intraoperative decision-making [16]. However, quantification requires stable ROI positioning relative to the target tissue throughout the measurement period, but this condition is rarely achieved in the dynamic surgical environment without motion compensation.

### 2.3. Data Acquisition and Region of Interest Selection (Annotation)

To evaluate motion compensation, video frames were systematically extracted from patient recordings at 10 s intervals, providing approximately 12–15 frames per case spanning 120–150 s of perfusion assessment. This interval was selected to capture the full dynamics of the ICG bolus, including arterial inflow and venous outflow while maintaining manageable data volume.

**Figure 2:**
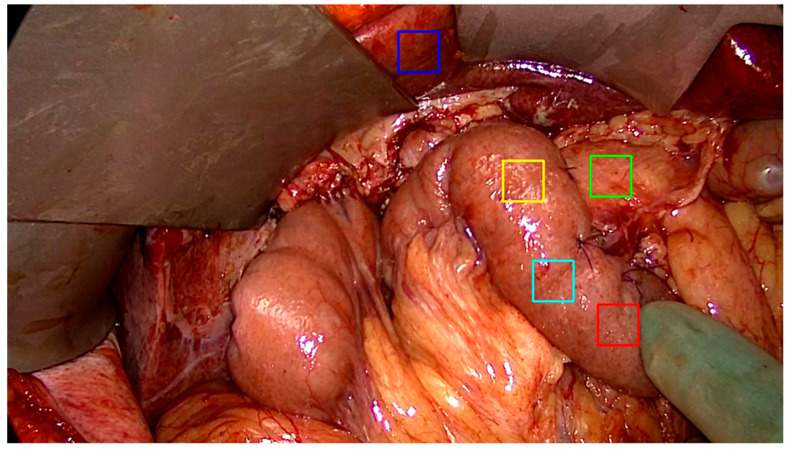
A view of the upper abdomen after resection and reconstruction of gastrointestinal continuity during a pancreaticoduodenectomy. Regions of interest (ROI) are placed on each frame, in a standardized fashion on the pancreas (green), oral jejunum (red, cyan, yellow), and liver (blue). These anatomical landmarks are selected due to their relevance to anastomotic integrity of the procedure. The blue liver ROI is selected as a reference point of fluorescence intensity in the frame series.

At each selected frame, five consecutive ROIs were placed by a single surgeon, in the same order, on a recognizable part of the organ. This provided a time series of ROIs placed by a surgeon for each procedure. ROI placement followed a standardized protocol: one ROI on the liver (serving as a reference organ with robust perfusion), one on the pancreatic remnant at the anastomotic site (the critical tissue of interest), and three on the jejunum (representing the conduit tissue). These anatomical landmarks were selected for their clinical relevance to anastomotic integrity and their distinct perfusion characteristics. Figure 2 illustrates the standardized ROI placement used throughout the study.

### 2.4. Motion Compensation

**Figure 3:**
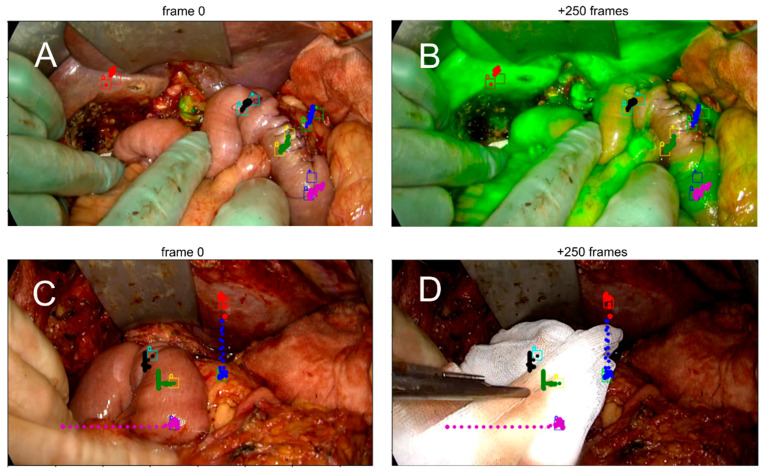
Demonstration of motion compensation (MC) during a fluorescence-guided pancreaticoduodenectomy. Each pair of images shows a region of interest (ROI) tracked over 250 frames. The colored dots indicate the MC path, while the squares mark manually annotated ROIs by the surgeon. (**A**,**B**) Adequate motion compensation, as judged by alignment between ROI position and MC trajectory despite physiological tissue movement. (**C**,**D**) Insufficient compensation due to external mechanical disturbance, resulting in a mismatch between the ROI and the MC path.

A motion compensation algorithm was developed using Python 3.12.2. (Python Software Foundation, 2024. Beaverton, OR, USA). The primary requirements for the algorithm, was that it should run in real time on a tablet [16], and that it should function during the significant change in appearance of the image during ICG inflow (Figure 3A,B). To achieve this, the final algorithm utilizes Phase Correlation (frequency-domain analysis) rather than standard intensity-based optical flow. This method identifies spatial translation by analyzing the spectral content of the image within a 150 × 150 pixel monitoring region surrounding each ROI. This subdivision minimizes the computational load. By relying on phase information rather than absolute pixel intensity, the tracking remains stable despite the changing fluorescence magnitude. Features that remain stable despite inflow of fluorescent dye include tissue edges, vascular patterns, and texture variations that remain relatively stable despite changing fluorescence intensity.

The algorithm calculates displacement vectors between sequential frames by computing the normalized cross-power spectrum, allowing it to predict how each ROI should move to stay in alignment with the initial target tissue.

The displacement vectors allow it to predict how each ROI should move to stay alignment with the initial target tissue.

### 2.5. Motion Compensation Evaluation

We wished to bridge quantitative image analysis and surgical assessment, providing a framework for integrating algorithmic feedback into intraoperative decision-making and for validating motion compensation as a clinically reliable tool in fluorescence-guided perfusion assessment. Thus we needed contemporary assessment by the computer and the surgeon.

The surgeon who placed the ROIs subjectively evaluated whether the motion compensation (MC) was adequate. This yielded a dataset of video sequences categorized into three MC performance levels: sufficient (1), insufficient (0), and failed (−1). Performance was classified as sufficient when the algorithm accurately tracked tissue movement in accordance with the surgeon’s manual adjustments, effectively compensating for motion. For each frame series, a comprehensive set of image quality and similarity metrics was computed to quantify how effectively tissue motion was corrected. Contrast and acutance characterized edge sharpness and visual clarity, and these features influence the surgeon’s ability to interpret perfusion. Local entropy measured texture complexity and information content within each ROI. Structural Similarity Index (SSIM) and Mean Squared Error (MSE) captured overall frame-to-frame alignment, while mutual information and delta entropy quantified temporal consistency and information retention after correction.

All metrics were standardized and analyzed using Principal Component Analysis (PCA) to uncover patterns distinguishing successful from unsuccessful motion compensation [25]. PCA reduces the dimensionality of the data while preserving variance, allowing visualization of the separation between sufficient and insufficient compensation cases. Receiver Operating Characteristic (ROC) curve analysis was performed to determine the discriminative ability of the PCA model, with the Area Under the Curve (AUC) providing a measure of overall performance.

Metrics were averaged across ROIs to yield representative value per series. Missing values were mean-imputed, and standardization completed using the StandardScaler from scikit-learn [26] to ensure equal weighting of features

### 2.6. Clinical Relevance

To assess the clinical relevance of motion compensation (MC), key perfusion parameters were compared with and without MC correction. Quantitative values were derived from time–intensity curves (Figure 1). Two representative parameters were selected for analysis: slope ingress (SLP), describing the rate of fluorescence increase during arterial inflow, and time-to-peak (TTP), representing the interval from the first fluorescence signal to maximum intensity. These parameters were chosen because they directly reflect tissue perfusion dynamics and have demonstrated correlation with clinical outcomes in previous studies [17]. By comparing SLP and TTP values before and after motion compensation, we evaluated whether MC improved the accuracy and stability of quantitative perfusion assessment. Both population-level and individual-level effects were analyzed to distinguish between overall bias and per-case changes in perfusion metrics.

### 2.7. Statistical Analysis

Statistical evaluation included both parametric and non-parametric analyses to comprehensively agreement, variability, and equivalence between compensated and non-compensated measurements. At the population level, paired t-tests were used to compare mean values of SLP and TTP before and after MC. To quantify agreement and bias while minimizing sensitivity to near-zero values, Bland–Altman analyses were performed using the signed relative difference (SRD = 2 × (Raw − MC)/(Raw + MC)), which symmetrically scales the difference by the average of the two measurements. The mean bias, standard deviation, and 95% limits of agreement were calculated for each metric. The geometric mean ratio (GMR) and corresponding 95% confidence intervals were calculated on log-transformed ratios (MC/Raw) to evaluate systematic proportional changes between methods.

Because motion correction could introduce asymmetric or non-normally distributed effects, Wilcoxon signed-rank tests were used to assess whether the median absolute percent change exceeded a ±10% tolerance. A Two One-Sided Test (TOST) for equivalence was additionally applied to test whether mean log-ratios fell within predefined ±10% bounds, considering it a formal test for practical equivalence between the two methods.

To evaluate directional and categorical agreement, a McNemar test was applied using a ±10% threshold, classifying measurements as “within tolerance” (|Δ| < 10%) or “changed” (|Δ| ≥ 10%), quantifying how often motion compensation induced substantial parameter shift.

All statistical analyses were performed in Python (SciPy v1.13).

## 3. Results

### 3.1. Summary

From the entire study cohort of 100 recordings, 17 randomly selected recordings were identified and enrolled for analysis. This included 12–15 extracted frames from each patient recording, corresponding to a recording length of 120 to 150 s segment of perfusion assessment. For visualization purposes, Figure 2 illustrates the initial placement of ROIs on each frame. After MC processing, 67.5% of frames were categorized as having correct and sufficient adjustment of ROIs when checked by the rater. Insufficient compensation occurred with the motion leading the tissue of interest outside the field of view, or when objects otherwise interrupted the view for extended periods, leading the algorithm to be incapable of performing corrective measures.

Videos were processed for analysis at a framerate of 41.5 frames per second, using a single CPU and ~220 mb of memory. Recording framerate was 25–30 frames per second.

**Figure 4:**
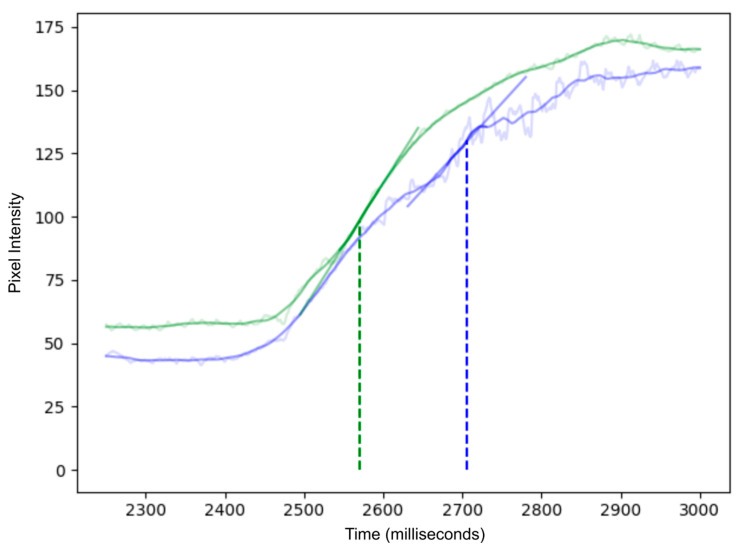
Time–intensity curves of ICG inflow with (green) and without motion compensation (blue). The raw fluorescence signal measured is shown in the lighter background trace for each curve, while the solid color curve represents normal smoothing algorithms applied in q-ICG, and these do not fully compensate for the artifacts. In the uncompensated (blue) curve, organ movement causes large fluctuations in the region of interest, leading to inaccurate perfusion measurements. In contrast, the motion-compensated (green) curve remains smoother, as the ROI tracks the target and eliminates motion artifacts. Linear regression lines at the point of greatest slope (indicated by vertical dotted lines) illustrate how motion compensation yields more reliable perfusion metrics.

Figure 3 compares representative examples of ROI tracking between sufficient and insufficient MC. The resulting difference in slope ingress (SLP) measured by quantitative ICG (q-ICG) analysis is shown in Figure 4.

**Figure 5:**
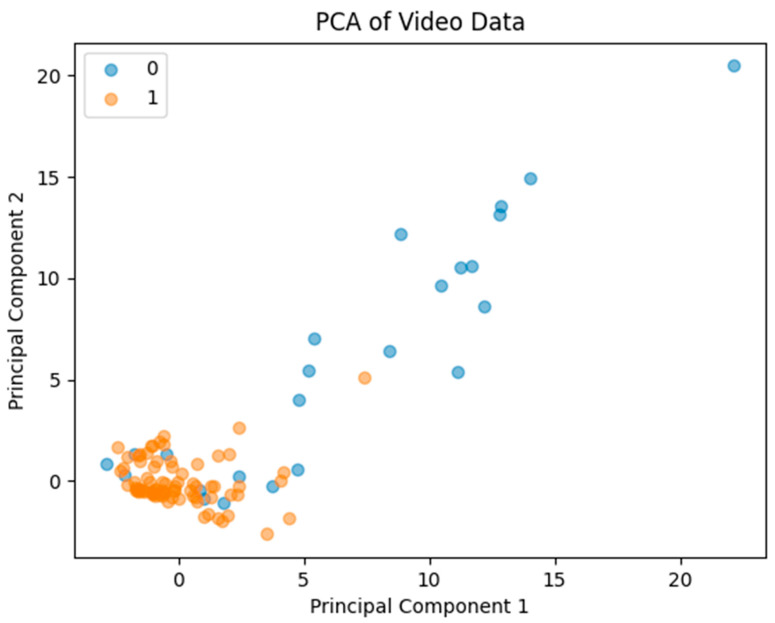
Principal Component Analysis scatter plot illustrating clear separation between sufficient and insufficient impact of MC. Area Under the Curve was calculated to be 0.8, confirming classification capability.

The PCA resulted in a reduced-dimensional representation of the metrics, capturing the majority of the variance in the first two principal components. Scatter plots revealed a distinct separation between the ‘sufficient’ and ‘insufficient’ categories, indicating that the MC algorithm had a noticeable impact on the metrics derived (Figure 5). PCA demonstrated clear separation between sufficient and insufficient MC cases, with an AUC of 0.8 confirming robust classification capability (Figure 5).

### 3.2. Implications on Perfusion Measurements

#### 3.2.1. Slope

Across 60 paired measurements, motion correction (MC) did not produce a statistically significant difference in perfusion slope values compared to raw data at the population level (paired *t*-test, *t*(59) = 1.60, *p* = 0.11), and neither did the geometric mean ratio (GMR) of MC to Raw slopes, which was 0.75 (95% CI 0.56–1.01) and not significantly different from unity (*t* = −1.92, *p* = 0.060), and therefore it did not meet the ±10% equivalence criterion (TOST *p* = 0.89).

**Figure 6:**
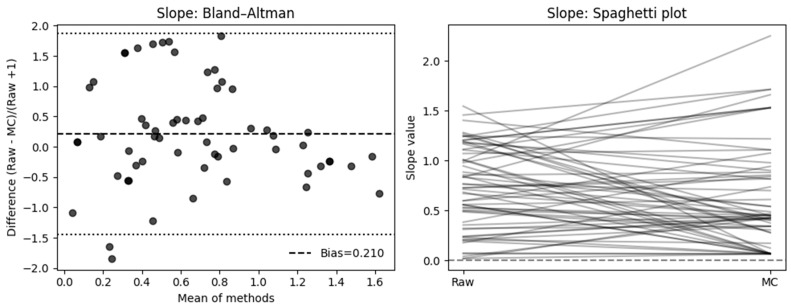
Effect of motion compensation on quantitative perfusion indicated by Slope during fluorescence-guided pancreaticoduodenectomy. Comparative analysis of the slope ingress of the time–intensity curve with and without MC. Bland–Altman plots demonstrate that MC significantly changed slope values and high variability (**left**). The right side shows a spaghetti plot, illustrating the individual changes in the ROIs before (Raw) and after motion correction (MC).

The SRD-based Bland–Altman analysis showed a modest positive bias (mean = 0.210, SD = 0.846, 95% CI [−0.004 to 0.424]), with wide limits of agreement (−1.449 to 1.869), reflecting substantial variability between Raw and MC measures. Confer Figure 6.

And the individual level, when expressed as percentage change relative to the raw measurement, 50% of cases showed a decrease ≥ 10%, 36.7% an increase ≥ 10%, and only 13.3% remained within ±10% of their original value. The mean absolute percent change was 108.5% (median = 37.8%, IQR = 20.2–81.9%), indicating considerable variability in slope measurements following motion correction.

A Wilcoxon signed-rank test of absolute percent change confirmed that median deviation exceeded the 10% tolerance (*p* = 1.00).

In a McNemar analysis classifying results by absolute deviation, 52 of 60 cases (87%) exceeded a ±10% change threshold, favoring the MC group, while only 8 (13%) remained within ±10% (χ^2^ = 30.82, *p* < 0.001).

#### 3.2.2. Time-to-Peak

Across 59 paired measurements, motion correction (MC) did not produce a statistically significant difference in time-to-peak (Tmax) values compared to raw data at the population level (paired *t*-test, *t*(58) = 0.81, *p* = 0.42). Likewise, the geometric mean ratio (GMR) of MC to Raw Tmax was 1.27 (95% CI 0.91–1.78), which was not significantly different from unity (*t* = 1.40, *p* = 0.17) and did not meet the ±10% equivalence criterion (TOST *p* = 0.80).

The Bland–Altman analysis using the signed relative difference (SRD = 2 × (Raw − MC)/(Raw + MC)) showed no evidence of systematic proportional bias (mean bias = 0.154, SD = 1.124, 95% CI [−0.131 to 0.438]). The limits of agreement (−2.050 to 2.357) suggest that individual measurements can differ by up to roughly twofold between the Raw and MC methods.

**Figure 7:**
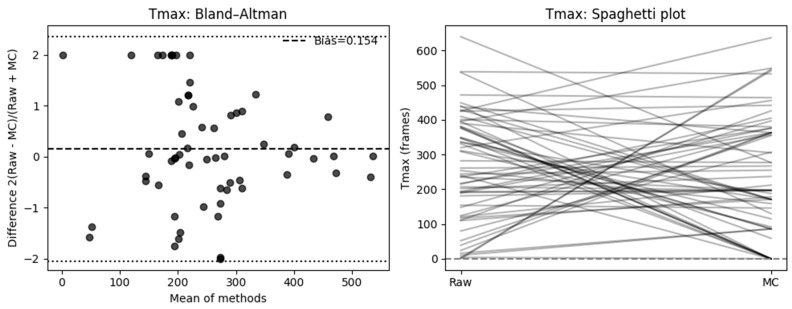
Effect of motion compensation on quantitative perfusion indicated by Slope during fluorescence-guided pancreaticoduodenectomy. Comparative analysis of the slope ingress of the time–intensity curve with and without MC. Bland–Altman plots demonstrate that MC significantly changed slope values and high variability (**left**). The right side shows a spaghetti plot, illustrating the individual changes in the ROIs before (Raw) and after motion correction (MC).

At the individual level, when expressed as percentage change relative to the raw measurement, 42.4% of cases showed a decrease ≥ 10%, while 37.3% showed an increase ≥ 10%, and 20.3% remained within ±10% of their original value. The mean absolute percent change was 431.5% (median = 65.9%, IQR = 19.6–100.0%), reflecting pronounced variability in Tmax measurements following motion correction. Cf Figure 7.

A Wilcoxon signed-rank test of absolute percent change confirmed that the median deviation exceeded the 10% tolerance (*p* = 1.00).

In a McNemar analysis classifying results by absolute deviation, 47 of 59 cases (80%) exceeded a ±10% change threshold, favoring the MC group, while only 12 (20%) remained within ±10% (χ^2^ = 19.59, *p* < 0.001).

## 4. Discussion

It is possible, given the right circumstances, to apply motion compensation (MC) effectively in quantitative ICG perfusion imaging (q-ICG), and it has a considerable effect on how perfusion results are interpreted. The current study demonstrated that it is both technically feasible and effective to apply MC for intraoperative perfusion assessment using q-ICG. Our analyses show that while MC significantly reduced artifacts and improved the visual stability of perfusion sequences, its impact on the quantitative parameters of perfusion was more complex.

### 4.1. Quantitative Effects of Motion Compensation

For perfusion slope, MC did not result in a statistically significant difference at the population; however, the relative Bland–Altman analysis indicated a mean bias of 0.21 (SD = 0.846, 95% CI −0.004, 0.424], LoA: [−1.449, 1.869]), suggesting a consistent change in slope after MC and high variability. At the individual level, 50% of cases showed a decrease ≥ 10%, 36.7% an increase ≥ 10%, and only 13.3% remained within ±10% of their original value. Neither the Wilcoxon signed-rank test (*p* = 1.00) nor the TOST equivalence test (±10% range, *p* = 0.89) supported equivalence between methods, which is supported by a McNemar analysis showing 87% of slope measurements differed by ≥10% (χ^2^ = 30.82, *p* < 0.001). For time-based perfusion quantification, motion correction similarly did not yield a significant mean difference. On an individual basis, 42.4% of cases showed a ≥10% decrease, 37.3% an increase ≥10%, and only 20.3% remained within ±10%. The mean absolute percent change reached 431.5% (median = 65.9%, IQR = 19.6–100.0%), indicating substantial variability in time-to-peak estimation following MC. A Wilcoxon test (*p* = 1.00) confirmed that median deviation exceeded the 10% tolerance, and McNemar analysis identified 80% of cases as exceeding this threshold (χ^2^ = 19.59, *p* < 0.001).

### 4.2. Interpretation and Implications

Collectively, these findings indicate that motion compensation significantly alters individual perfusion parameters, even when average changes at the population level appear nonsignificant. This makes sense, as motion can lead to artifacts in both directions. The absence of equivalence within ±10% for both slope- and time-based underscores that MC introduces meaningful numerical shifts, which—while mitigating artifacts—affect the quantitative reproducibility of q-ICG measurements. From a clinical perspective, this is crucial. The inequivalence of perfusion metrics observed in the motion-compensated sequences implies that motion artifacts may previously have led to biased slope estimates of perfusion rates. Such distortion can alter surgical decision-making, particularly when assessing tissue viability along delicate resection margins. When millimeters of viable tissue may determine whether a section is preserved or removed, even small deviations in perfusion metrics could influence clinical judgment.

Inadequate perfusion may have severe clinical consequences, including the risk of anastomotic leakage and progression of ischemic tissue [27]. By eliminating fluctuations on ICG time–intensity curves caused by physiological movements, MC improves the accuracy of the perfusion assessment and thus becomes more reliable as an adjunct for viability assessment. Our results are consistent with work in other surgical fields, indicating a broad applicability of this technology. In one orthopedic study of 18 amputation cases, surgeons applied MC to q-ICG assessment of periosteal perfusion [28]. Their results demonstrated that only after MC was applied, were the expected differences in perfusion parameters observed (maximum intensity, SLP, and TTP). Similar applications could extend to vascular surgery following revascularization [29], flap viability evaluation [30], and anastomotic perfusion verification in colorectal surgery for [3,21,22,23], all of which rely on accurate quantification of perfusion data to identify outcomes. This consistent finding indicates that MC addresses a universal challenge in fluorescence-guided surgery.

The potential for standardization represents another significant advantage of automated MC. Currently, fluorescence-guided surgery has considerable interobserver variability [12,31], and interpretation of the fluorescent signal is dependent on previous experience [32]. For colorectal surgery, q-ICG is feasible but has yet to be proven clinically superior to qualitative ICG [33]. Motion compensating for artifacts and thus enhancing perfusion assessment draws q-ICG closer towards evaluating its effect on clinical outcomes in a robust fashion. By minimizing the variability introduced by motion artifacts, MC provides more consistent and reliable measurements regardless of the assessor. Standardizing perfusion criteria would support universal thresholds for tissue viability and enable evaluation of outcomes through clinical trials, representing a gap in current practice.

A strength of our approach is the practical implementation and ability to assess perfusion by a handheld optic device. Unlike many technological advances that demand substantial equipment upgrades or disrupt established workflows, our MC algorithm requires minimal computational resources and can be seamlessly integrated into existing surgical imaging systems. The low processing requirements allow surgeons to receive feedback on perfusion assessment within seconds, which is a critical advantage in acute care surgery where time is essential. Furthermore, integration would require minimal training since the system automatically tracks tissue movement beyond the initial ROI placement.

Despite these promising results, several limitations must be acknowledged. Different or better performing motion correction could lead to different results, but in our situations the algorithm could correct ROI placement satisfactorily in 67.5% of the frame series. The remaining situations with blocking of original ROI placement and exaggerated motion are not correctable using our method. Examples of these are tissue wiping, considerable camera movements where the target leaves the field of vision, or manipulation by surgical instrument. Thus, where combination of recording and initial ROI placement leads to the algorithm incapable of achieving good measurement. The PCA-based evaluation was a further attempt to enable feedback on the trustworthiness of the data, it suggests that MC performance can be optimized through threshold calibration, supporting the feasibility of integrating MC directly into imaging systems for real-time feedback on signal stability.

Lastly our study focused exclusively on pancreaticoduodenectomies, which does not represent all surgical settings where fluorescence guidance is valuable. These limitations suggest that procedural adaptations (for instance, minimizing tissue manipulation during critical measurement periods and maintaining a relatively stable camera positioning) are necessary to maximize the benefits of MC. Based on these realizations, future improvement efforts could focus on further technical development to ease usability before exploratory pilot studies can evaluate clinical feasibility. In future work, multicenter validation studies may be feasible, as the proposed motion compensation approach is independent of the specific surgical procedure and relies solely on image-based features derived from fluorescence recordings. However, successful implementation across centers would require consideration of variability in surgical workflows, differences in surgical optical equipment and acquisition parameters, and variations in tissue perfusion characteristics across organs (e.g., ventricle versus intestine) and surgical domains. This knowledge and experience with fluorescence-guided surgery is essential when designing broader validation studies. Additionally, the high variability observed in time-to-peak measurements indicates that motion compensation may not uniformly enhance all aspects of perfusion assessment. It remains to be determined whether this variability was due to limitations of the compensation algorithm or reflects genuine biological differences in perfusion dynamics, given that the true perfusion values were unknown. Future studies should correlate MC-enhanced perfusion measurements with clinical outcomes to conclusively establish its impact on complication rates, particularly regarding anastomotic leakage.

In conclusion, this study demonstrates that MC has a substantial impact on proper quantitative perfusion assessment with ICG, and is necessary if using q-ICG as a viability assessment adjunct. By effectively correcting motion artifacts, MC significantly improves the accuracy of fluorescence-based evaluation at the organ of interest. MC should be considered essential for an adequate perfusion evaluation. The technology’s low computational requirements and its compatibility with existing surgical workflows render it particularly valuable for immediate clinical implementation, offering the potential to standardize perfusion assessment, facilitate inter user reproducibility and evaluate its effect on clinical outcomes.

## Data Availability

The datasets presented in this article are not readily available because they contain sensitive personal data, cf. EU GDPR, and cannot be shared without consent. Requests to access the datasets should be directed to the corresponding author.
